# Recent Advancements in Understanding Primary Cytomegalovirus Infection in a Mouse Model

**DOI:** 10.3390/v14091934

**Published:** 2022-08-31

**Authors:** Kimberley Bruce, Jiawei Ma, Clara Lawler, Wanxiaojie Xie, Philip G. Stevenson, Helen E. Farrell

**Affiliations:** School of Chemistry and Molecular Biosciences, University of Queensland, Brisbane, QLD 4072, Australia

**Keywords:** mouse cytomegalovirus, olfactory epithelium, herpesvirus spread, dendritic cells, viral G protein-coupled receptor, animal model

## Abstract

Animal models that mimic human infections provide insights in virus–host interplay; knowledge that in vitro approaches cannot readily predict, nor easily reproduce. Human cytomegalovirus (HCMV) infections are acquired asymptomatically, and primary infections are difficult to capture. The gap in our knowledge of the early events of HCMV colonization and spread limits rational design of HCMV antivirals and vaccines. Studies of natural infection with mouse cytomegalovirus (MCMV) have demonstrated the olfactory epithelium as the site of natural colonization. Systemic spread from the olfactory epithelium is facilitated by infected dendritic cells (DC); tracking dissemination uncovered previously unappreciated DC trafficking pathways. The olfactory epithelium also provides a unique niche that supports efficient MCMV superinfection and virus recombination. In this review, we summarize recent advances to our understanding of MCMV infection and spread and the tissue-specific mechanisms utilized by MCMV to modulate DC trafficking. As these mechanisms are likely conserved with HCMV, they may inform new approaches for preventing HCMV infections in humans.

## 1. Introduction

Human cytomegalovirus (HCMV) is the leading infectious cause of congenital abnormalities in children, with 200–600 babies born each year in Australia with symptomatic disease, that includes intellectual disability, neuromotor deficits, hearing and vision loss, microcephaly and hepatosplenomegaly [1]. Such harm is especially likely to arise from a primary maternal infection [2]. HCMV is transmitted via saliva, urine, and breast milk. Following HCMV infection, the virus becomes latent and capable of intermittent reactivation during episodes of immunosuppression. Vaccination against congenital HCMV infection has been a major public health goal for more than 50 years, but to date, no vaccine has achieved licensure [3]. Due to risk of latency and reactivation with live attenuated vaccines, strategies have focused on intramuscular inoculation of recombinant subunit formulations. Many such vaccines have proved highly immunogenic, but none have protected against congenital infection [4]. HCMV infection is highly cell-associated; in latently infected individuals, carriage is maintained by myeloid cells, particularly those of the dendritic cell (DC) lineage (reviewed by [5]). However, little is known of HCMV spread during primary infection, and given the correlation of primary infection with increased disease, understanding the natural bottlenecks that must be negotiated to achieve systemic spread will inform antiviral strategies.

HCMV infection is asymptomatic in immunocompetent hosts, so capturing the early events of a primary infection is difficult. Acquisition of HCMV early in life is common [6]. In Australia, approximately 38% of children aged under 2 years are seropositive for HCMV; a striking increase in the rate of seroconversion (to an average of 58%) occurs in women of childbearing age suggesting they acquire their primary infection from exposure to infected children. Given the rate of virus transmission to the placenta during primary infection is estimated at 30%, of which 11% result in a symptomatic HCMV-infected child at birth, the disease burden of congenital infection is significant. (Reviewed in [1]) HCMV transmission in breastfed newborns, daycare settings and PCR-positive oropharyngeal swab specimens taken from infants has been presented as evidence in support of oral HCMV entry [7,8,9]. However, cells infected in the oral mucosa have yet to be identified, and difficulties arise differentiating virus entry from virus exit in humans. As newborns are obligate nasal breathers in the first few months of life, there is potential for infected breastmilk and saliva to gain access to olfactory epithelium that line the nasal turbinate [10].

As with most beta-herpesviruses, CMV infections are highly species-specific and thus precludes analysis of HCMV colonization and spread by infection of experimental animals. Natural animal models include the use of mouse CMV, rat CMV, guinea pig CMV and rhesus CMV. Herpesvirus colonization of mammals preceded primate/rodent divergence; they evolved predominantly by co-speciation and therefore virus–host immune interactions have been largely preserved, despite loss of sequence conservation in the molecules that articulate such interactions [11]. The CMVs and deliberate infection of experimentally accessible mammals can thus provide authentic readouts. With respect to understanding the early events in host colonization and spread, cost and availability of biological tools and reagents makes the MCMV model a highly feasible choice for study. 

The MCMV model has been instrumental in understanding virus–host interactions and disease outcome; numerous seminal studies led by Australian scientists and international collaborators over the past 40 years. These contributions have been the subject of a comprehensive review [12]. Until recently, the model had not been exploited for understanding the early events of natural virus entry and spread. Like HCMV infection, the prevalence of multiple MCMV strains in the wild is high [13,14]. Yet, in the laboratory setting, MCMV transmission efficiency is generally poor and deliberate superinfection in laboratory mice seemed difficult to achieve [15]. Either the laboratory setting was deficient due to phenotypic differences between wild and lab-adapted viruses [16], or the animal husbandry methods used to facilitate transmission were lacking—or both. 

## 2. MCMV Uses Olfaction, an Ancient Vertebrate Sensory System, for Entry

**Figure 1:**
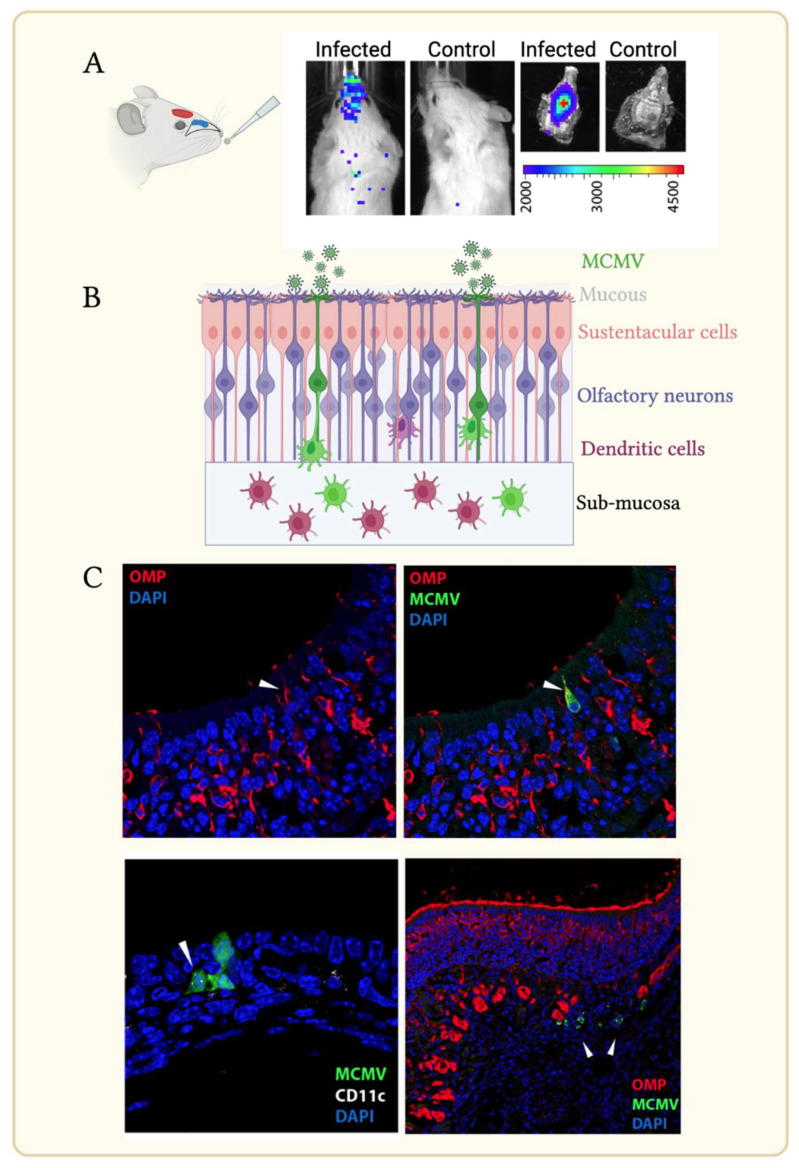
The olfactory epithelium is a site of MCMV entry. (**A**) Alert mice inoculated with <5 uL of inoculum to the nares. The olfactory epithelium (blue) lines the nasal turbinate protruding into the nasal cavity. Axons of olfactory neurons link to the olfactory bulb (red) which is positioned anterior to the brain. Nose infection detected by live imaging 3 days post-infection with a luciferase-tagged MCMV (**left**) compared with an uninfected control mouse (**right**); dissection of the palate reveals localized luciferase expression in infected animals. The light intensity scale (p/s/cm^2^/sr) is shown. (**B**) Simplified schematic of olfactory infection. Olfactory neurons, whose dendrites contact the environment above the mucous layer are the first targets for MCMV. Infection passes to sustentacular cells and dendritic cells (DC). Infected DC mobilize to the submucosa. (**C**) Immunohistochemical detection of infected of OMP^+^ olfactory neurons (upper panel, arrow) 24 h following infection with an EGFP-tagged MCMV; infection of an adjacent CD11c^+^ cell detected day 3 p.i. (lower panel left; arrow); by day 4 p.i. numerous infected cells presumed to be DC based on further staining (arrowed) were detected in the sub-mucosa (lower panel, right).

Previous studies in the Stevenson laboratory at the University of Cambridge had demonstrated olfactory colonization by HSV and MuHV-4, achieved by inhalation of <5 μL of virus inoculum from the nares of non-anaesthetized adult mice [17,18]. However, *Mus musculus* are not natural hosts for these herpesviruses and thus natural olfactory herpesvirus transmission remained to be demonstrated. Studies by the Nauwynck laboratory reported infection of the murine olfactory epithelium by high volume oronasal infection [19]. However, it was unclear if MCMV spread emanated from oral and/or nasal colonization. Alert mice inoculated with low volume luciferase-tagged MCMV (MCMV-luc), followed by longitudinal luciferase detection via unbiased whole-body imaging and tissue dissection confirmed olfactory colonization (Figure 1A) [20]. Deliberate olfactory inoculation of adult and neonatal mice with MCMV-luc resulted in successful colonization of the nose, detectable by 3 days post infection (p.i.), with spread to the superficial cervical lymph nodes and salivary glands over the following 1–2 weeks. While spread from the nose was asynchronous, all mice exhibited spread to the salivary gland and all seroconverted. Lung infection, achieved by larger inoculums delivered under light anesthesia, also spread via the draining mediastinal LN (mLN), with the advantage that spread was synchronous and therefore, predictable. Oral MCMV-luc infection did not show evidence of infection, nor evidence of seroconversion [20,21].

Successful MCMV infection by deliberate olfactory inoculation was one thing; demonstrating that it occurred naturally was quite another. The highest incidence of HCMV infection occurs in the first few years of life, acquired from infected breast-feeding mothers and infected peers [7,9]. In laboratory mice, transmission of MCMV infection from infected dams to pups was unsuccessful if breeders were offered loose bedding in standard laboratory cages to make shallow, exposed nests which the dams left frequently unattended. In contrast, provision of enclosed cardboard nests in undisturbed cages that encouraged natural maternal behavior-exemplified with prolonged contact between dams and pups-resulted in 80% transmission rate. All infected pups exhibited seroconversion. Prolonged exposure to infected mice thus likely overcomes the anatomical hurdle required for olfactory access, rather than inefficiency of MCMV spread once colonized [20].

Like many sensory receptors, olfactory receptors (OR) belong to a large and ancient superfamily of G protein-coupled receptors [22]. Olfaction is essential for numerous behaviors essential for varying facets of life; examples include food gathering, mating and predator avoidance. Bipolar olfactory neurons (ON; positive for the olfactory marker protein, OMP) interpose the supporting columnar sustentacular cells within olfactory epithelium, but their filamentous dendritic processes extend apically, with each ON dendrite comprising 10–15 immotile cilia, increasing the surface area for stimulus capture (Figure 1B). The cilia contain the odor receptors for conducting inhaled stimuli via GPCR and intracellular second messages to the olfactory bulb [23]. Cilia are bathed in mucous that is in contact with the environment; in terrestrial animals the mucous contains odorant binding proteins which are thought to localize, solubilize, and transport odor ligands to the olfactory GPCR. Sustentacular cells also possesses apical microvilli that intermingle with ON cilia within the mucous, but they do not extend beyond the base of the mucous layer [24]. The critical structures of the olfactory epithelium are monitored for infection by the ancient nasopharynx-associated lymphoid tissue (NALT). Studies in fish and mice have demonstrated close links between olfactory-mediated behaviors linked to activating the local immune system, including myeloid cells, microglia, and neutrophils [25,26]. CD68^+^ myeloid cells are found closely associated with ON and are important for their neuroprotection and neurogenesis [27]. Infection with an EGFP-tagged MCMV showed infection of OMP^+^ ONs, that extend their dendrites above the olfactory mucus (Figure 1B,C). Infection with a “single-cycle” MCMV mutant (i.e., being incapable of spread beyond the first infected cell) confirmed ONs to be the primary targets. Sustentacular cells seem secondarily infected, consistent with findings from the Nauwynck laboratory [19] (Figure 1C).

A feature of many herpesviruses is their ability to bind heparan [28]. Indeed, heparan binding is the initial step to cell entry for numerous viruses, such as SARS-CoV-2, papillomaviruses, and respiratory syncytial virus [29]. In vivo, mucosal access to heparan on most epithelia is prevented due to its basolateral expression; indeed, for papillomaviruses, epithelial abrasion, exposing heparan, is required for virus entry [30]. The olfactory epithelium is a notable exception, and it expresses heparan on its apical surface. [24,31]. Potentially, the olfactory epithelium provides a niche environment to accumulate and concentrate herpesviruses that are arrested by binding to heparan. Examples of olfactory entry come from all herpesvirus subfamilies, highlighting utilization of an anatomically conserved entry route that provides optimal capture [17,19,20,31,32]. In humans there is evidence supporting beta-herpesvirus colonization via olfaction: the human olfactory GPCR OR14I1, an olfactory sensor, is a receptor for HCMV [33].

Recombination is evident in the HCMV and MCMV genomes, possibly facilitating compensation for host recombination that affects transmission efficiency, particularly in countering host immunity [34,35,36,37]. Previous studies using invasive infections in laboratory mice exhibited extensive tissue co-carriage of genetically distinct MCMVs [38,39,40,41], but evidence for recombination was lacking. Non-synchronous olfactory infections of MCMVs with equivalent fitness in nose colonization but each attenuated for systemic spread via separate mutations in distinct loci gave rise to wild type-like recombinants with greater ability to spread than each inoculum. Thus, olfactory infection provided the optimal setting to promote selection of herpesvirus recombinants with improved fitness [42]. Interestingly, olfactory receptors exhibit rapid evolution in their repertoire following environmental change, including changes generated anthropomorphically; how such changes might drive HCMV evolution is unknown [43,44].

## 3. MCMV Infection Affects DC Directional Decision-Making to Facilitate Systemic Spread

Histological analysis of lymph nodes (LN) draining the nose and lung following lung or olfactory MCMV infection showed that MCMV^+^ cells expressed CD68^+^ and CD11c^+^; markers expressed by both dendritic cells (DC) and alveolar macrophages (AM) [45]. Intranasal delivery of PKH26 or dextran-conjugated Texas Red dyes (markers of phagocytosis and micropinocytosis, respectively) a day before MCMV lung challenge demonstrated that the infected cells mobilized to draining lymph nodes (LN) were highly endocytic, but poorly phagocytic, consistent with DC rather than AM. A distinguishing feature of MCMV^+^ DC is the punctate localization of the CD11c^+^ integrin. Confirmation of DC as the principal vehicle for MCMV transport came from CD11c-cre mice infected with a MCMV mutant tagged with a floxed GFP gene upstream of a nuclear-targeted td Tomato gene (designated MCMV-GR). MCMV-GR infection in CD11c^+^ cre^+^ cells irreversibly switched infected cells from green to nuclear red fluorescence. While less than 25% of infected cell in the lung mucosa exhibited color switching at day 1 p.i., 80% of MCMV^+^ cells detected in the draining MLN were color-switched CD68^+^ CD11c^+^ cells, demonstrating preferential mobilization of CD11c^+^ infected cells. In the blood, MCMV genomes were found concentrated in CD11c^+^-purified fractions [45].

The trafficking of activated DC from non-lymphoid peripheral sites to draining lymph nodes via engagement of CCR7 and CCL21/19 has been well described [46]. Expression of CD44 is also required for the homing of DC to LN and their subsequent trafficking with LN to T cell zones. DC use fibroblast reticular cells (FRC; marked by antibodies to the stromal ER-TR7^+^ fibers) as conduits within the LN parenchyma for optimal positioning near high endothelial venules (HEV) for antigen presentation. HEVs continually recruit naïve and memory lymphocytes from the bloodstream for the purpose of immune surveillance; they express the sialomucin peripheral lymph node addressin (PNAd; marked by antibody MECA-79 [47]) which facilitates blood-borne lymphocyte arrest as the first step to LN entry [48,49]. 

**Figure 2:**
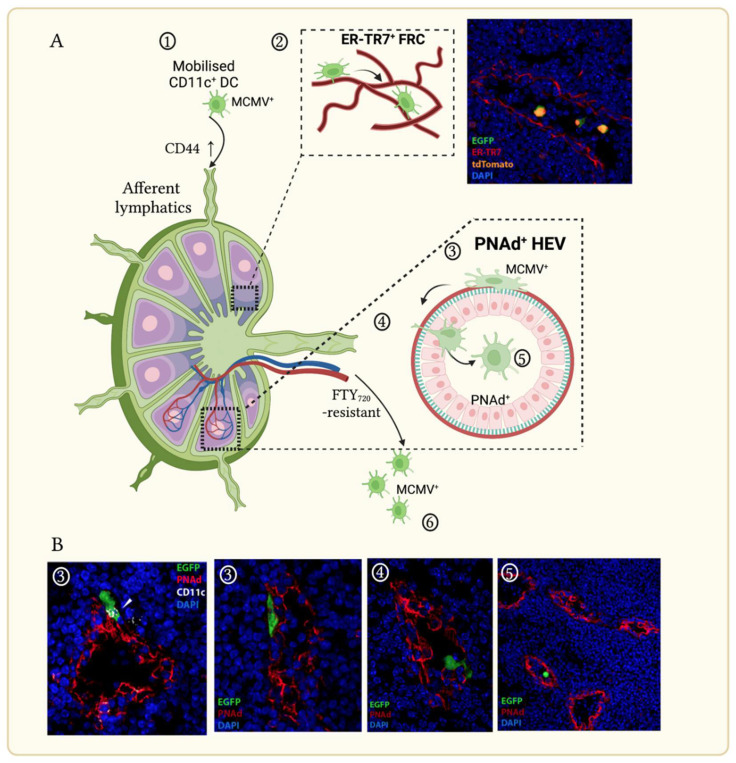
MCMV modulates dendritic cell (DC) directional decision-making in draining lymph nodes (LN). (**A**). Schematic showing MCMV-infected DC entry to LN draining the olfactory or lung mucosa via afferent lymphatics. (1) MCMV^+^ DC traffic via afferent lymphatics, facilitated by CD44. (2) CD11c-cre mice infected with a MCMV mutant possessing a floxed EGFP upstream of a nuclear-localized td-Tomato exhibit color-switched CD11c^+^ cells infiltrating the LN via ER-TR7^+^ fibroblastic reticular cells. (3) MCMV^+^ CD11c^+^ DCs of mice infected with EGFP-tagged MCMV associate with PNAd^+^ high endothelial venules (HEV), then extravasate (4) to enter the HEV lumen via a mechanism resistant to fingolimod treatment (FTY_720_). (5) Infected DC escape to the blood. (6) (**B**). Immunohistochemical analyses of LN draining the olfactory or lung mucosa taken days 1–3 p.i. showing MCMV^+^ DC interaction with PNAd^+^ HEV with reference to schematic pathway indicated numerically in (**A**). Arrow denotes punctate CD11c^+^ expression by MCMV-infected DC.

Within hours following lung or olfactory infection, MCMV^+^ DC enter the draining LN (mediastinal or superficial cervical, respectively) via afferent lymphatics, colocalize with ER-TR7^+^ FRC in LN and traffic to the central, medullary region. In MCMV-infected immunocompetent hosts LN FRC do not appear to become infected (Figure 2A). Activated DC are presumed to die in LN, since efferent lymph contains few myeloid cells [50,51]. However, MCMV-infected cells did not accumulate in LN. CD11c^+^ cells were frequently located against the basolateral surface of PNAd^+^ HEV within hours following mucosal infection (Figure 2B). Up to 30% of MCMV^+^ DC in LN sections were associated with HEV 1–2 days post infection, with numerous examples of localization in the lumen [52]. No preferential positioning of MCMV^+^ DC was associated with LYVE-1, a lymphatic cell marker. CD44 expression facilitated MCMV^+^ DC trafficking, consistent with its expression on infected cells being unaffected by MCMV infection [53]. LN traverse and escape to the blood by MCMV^+^ DC were unaffected by treatment of mice with fingolimod (FTY_270_)—an inhibitor of the sphingosine-1-phosphate receptor-1 (SIPR1) that facilitates LN lymphocyte egress via efferent lymphatics [54]. Taken together, the results of these longitudinal studies demonstrated that while MCMV-infected DC retain the ability to traffic to draining LN, they are driven to escape to the blood via HEV using a mechanism resistant to established LN retention signals [45].

Thus, viremia following lung or olfactory infection is comprised almost exclusively of infected CD11c^+^ DC; less than 2% of the viral genomic load is found contained in cell-free plasma. MCMV-infected CD11c^+^ cells extravasated widely to numerous tissues, and while the viral load sustained in peripheral tissues was low (with exception of the salivary gland where it is subsequently amplified in acinar epithelial cells), it is nevertheless reactivatable from latency months late [40,55]. DC colonization of the salivary gland is detected by day 4 post mucosal challenge; lung DC infected with a single-cycle MCMV mutant, incapable of cell–cell spread are readily detected in the SG, confirming DC mobilization all the way from the site of viral entry to exit [45]. This silent, yet efficient asymptomatic spread mimics HCMV infection in humans.

## 4. The MCMV Encoded Chemokine Receptor M33 Drives DC-Dependent Spread via Tissue-Specific Signaling Mechanisms

All beta-herpesviruses possess homologues of seven transmembrane-spanning GPCRs which have been categorized into three conserved gene families, designated UL33, UL78 and US28 (in reference to the HCMV ORFs) [56]. UL33 and UL78 gene families are conserved in all beta-herpesviruses; the US28 gene family is a more recent capture by primate herpesviruses genomes. All beta-herpesvirus GPCR (vGPCR) possess a transmembrane III “DRY” motif (or conserved derivative) that is important for GPCR stabilization, and all vGPCR tested to date are constitutively endocytosed. HCMV US28, UL33 and CMV counterparts exhibit constitutive signaling and as they are present on the virion, they have the potential to impose intracellular activation upon virus entry [57,58]. Their pathway repertoires resemble onco-modulatory signaling “signatures” [59]. US28 and UL33 vGPCR families bear homology to CC chemokine receptors; US28 binds multiple chemokines—even across the chemokine CC, CXC and CX3C classes and this promiscuity allows for differential control to signaling output and cellular function. Moreover, US28 engages multiple G proteins: G_αq_, G_αi/o_ and G_α12/13_ which appear to be important in cellular mobilization in different cellular contexts in vitro. HCMV UL33 and its CMV counterparts remain orphan receptors, although the HHV-6 UL33 homolog binds CCL2–5 chemokines. (Reviewed by [60]) Constitutive signaling by UL33 in transiently transfected trophoblasts promotes their mobilization and its absence attenuates cell-free and cell–cell spread in vitro [61,62].

**Figure 3:**
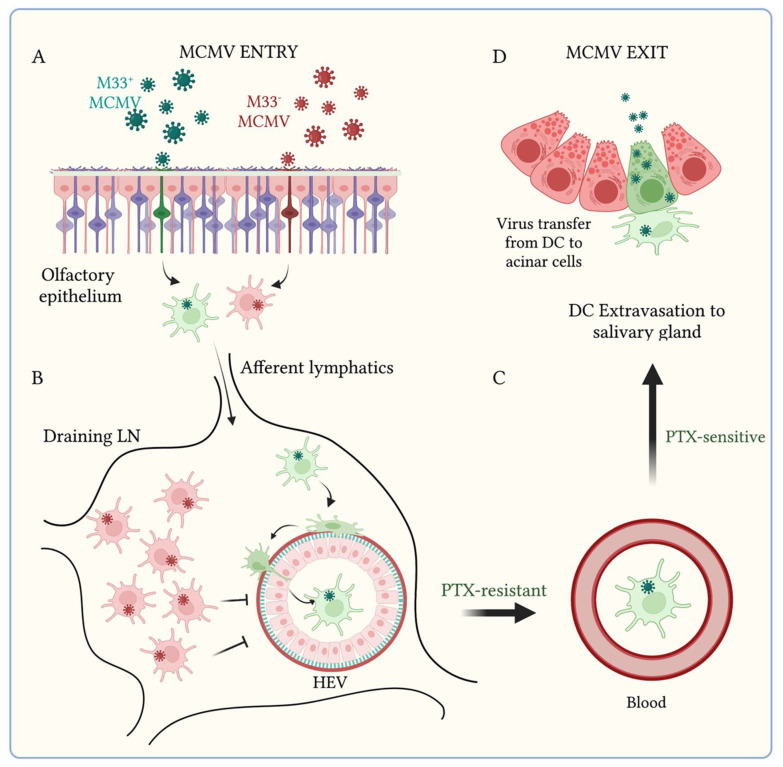
The MCMV encoded chemokine receptor M33 drives DC-dependent systemic spread via tissue-specific signaling mechanisms. Schematic showing the fate of M33^+^ and M33^−^ MCMV infections in vivo. (**A**) Mucosal infections at the olfactory or lung mucosa with either M33^+^ (green) or M33^−^ MCMV (red) spread via the afferent lymphatics to draining LN, facilitated by CD44 (**B**). DC infected with M33^+^ MCMV traffic to HEV and escape to the blood (**C**) via a mechanism resistant to pertussis toxin (PTX). In contrast, DC infected with M33^−^ MCMV show reduced association with HEV and viraemia and instead accumulate in LN. Blood-borne MCMV M33^+^ DC extravasate from the blood to the salivary glands via a PTX-sensitive mechanism, where genome amplification in acinar epithelial cells (green) precedes virus exit (**D**).

To date, deletion of UL33 homologs in rodent CMVs attenuates salivary gland infection [40,63,64,65]. Tracing virus spread following intranasal infection of mice with a knockout of the MCMV UL33 homolog, (ΔM33) revealed diminished viremia compared with M33^+^ MCMV. ΔM33 MCMV^+^ DC accumulate in LN and exhibit reduced association with HEV (Figure 3). A single point mutation of the M33 TMIII “DRY” motif” that ablates constitutive G_αq_-dependent signaling confers the same in vivo attenuation [45]. Notably, the M33 knockout is rescued by HCMV US28 expression (i.e., infection with a MCMV M33^−^US28^+^ mutant) but not by a signaling null US28 DRY^−^ counterpart. Thus, LN traverse and DC escape to the blood is dependent on the constitutive vGPCR signaling conserved between M33 and US28 [52]. While vGPCR constitutive activity alone may account for biological function of M33, the possibility remains that its engagement with a cognate ligand by M33 (and conserved in US28) may be important for driving trafficking of infected DC. 

The rescue of M33 by US28 suggested that US28 may provide a similar function in HCMV-infected DC. However, while US28 could rescue M33 with respect to viremic spread, it was unable to rescue salivary gland infection [52]. Thus M33 has a second essential function: to facilitate DC extravasation from the blood to the salivary gland. This additional role was confirmed by analysis of the systemic dissemination of a 38 amino acid truncated carboxy-tail M33 mutant (M33ΔC38) for which G_αq_-dependent CREB activation was disabled, but PLC-β signaling at the cell membrane was preserved [55]. Intranasal infection resulted in arrest of M33ΔC38-infected DC in the draining lymph nodes, similar to ΔM33, demonstrating that PLC-β signaling here was redundant. However, when MCMV-infected DC were delivered directly to the bloodstream via intravenous transfer, M33ΔC38 was competent for extravasation to the salivary glands, but this was mediated by a G_αi/o_-dependent, and CREB-independent mechanism. Thus, MCMV M33 temporally orchestrates DC trafficking by engaging different G proteins in different tissues. Olfactory signal transduction is mediated by GPCRs [66]. While M33 does not appear to be responsible for MCMV entry at the olfactory epithelium in the laboratory setting, there is potential for MCMV GPCRs to interfere with olfaction. 

## 5. A MCMV Chemokine Homolog Facilitates Virus Infection of Salivary Gland Acinar Cells

Both HCMV and MCMV possess a C-C chemokine homolog, designated UL128 and m131/129 (more commonly known as MCK2), respectively [67,68,69]. Apart from chemokine motifs, there is little interspecies sequence conservation, although they share positional homology in their respective genomes. Both HCMV and MCMV C-C chemokine homologs are integral constituents of viral tropism complexes comprised of glycoproteins H and L [70]; in HCMV this complex also includes adjacent gene products UL130/131A [71].

**Figure 4:**
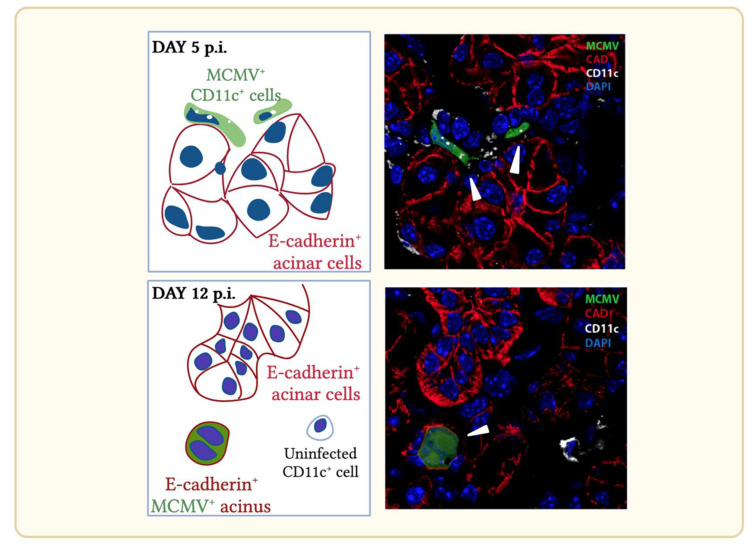
Schematic and corresponding immuno-histochemistry of cells infected in the salivary gland during acute (day 5) and persistent (day 12) MCMV infection. Mice infected intranasally with EGFP-tagged MCMV exhibit infected cells at day 5 p.i. and display characteristic punctate CD11c expression. Infected cells lie interposed between E-cadherin^+^ salivary gland acinar cells. By day 12 p.i. wild type MCMV infection is found predominantly in CD11c^-^/E-cadherin^+^ acinar cells. Infected cells indicated by arrows.

In vivo, MCMVs deleted of m131/129 exhibit reduced infection in the salivary gland [39,72]. Tracking virus spread from the olfactory epithelium revealed equivalent colonization by day 3 p.i. and spread to the salivary glands. However, amplification in the salivary glands by Δm131/129 MCMV was reduced thereafter. Immunohistochemical analyses of cells infected in the salivary glands revealed that CD11c^+^ cells infected with Δm131/129 MCMV did not transfer infection efficiently to aquaporin^+^ cadherin E^+^ acinar cells (Figure 4). Dissemination events upstream of the salivary gland, are not dependent on m131/129 [73]. The mechanism of m131/129-dependent transfer from DC to acinar cells is not yet understood, but it may reflect its association with the fusogenic glycoprotein H/glycoprotein L (gH/gL) complex promoting cell–cell spread [73]. 

## 6. Conclusions

In recent years, we and others have identified olfactory colonization of mice with MCMV and DC-dependent spread [19,20,45]. Olfaction is an ancient sensory system and more than 350 million years old in vertebrates. Canonical DC have a shared ancestry with similar timescale [22,74]. Both developed before the ancestral herpesvirus diverged into the three herpesvirus subfamilies (180–220 Ma) [11,75]. Host changes will drive virus change in species sub-lineages, but these are likely to be compensatory measures to preserve mechanisms of colonization and spread. Therefore, it is reasonable to predict that HCMV also uses olfaction to gain entry. 

Tracking MCMV infection has demonstrated focused infection of the olfactory epithelia which is spread efficiently and silently by DC. These characteristics mimic asymptomatic myeloid-specific spread described for HCMV. Even when MCMV infection is amplified in the salivary glands, the number of infected cells is unexpectedly low. Opportunities for MCMV recombination are thus limited. Since it is unclear how MCMV recombinants with improved fitness for spread could be selected at the point of host exit, the most likely site for recombination thus lies with the olfactory epithelium. In humans, close interactions in the early years of life—with parents, siblings, day-care and later during adolescence provides the most likely settings for sequential infections. 

To date, no vaccine has been developed with sufficient efficacy in preventing congenital infection to warrant licensure. Infected DC are put center stage in key dissemination bottlenecks: at the olfactory epithelium, the draining lymph node, and extravasation to salivary gland acinar cells. Thus, DC are important targets in mitigation design. In MCMV, olfactory vaccination with a mutant deficient in M33 which limits DC-dependent spread showed protection against systemic spread following superinfection, supporting a DC-targeted approach to intervention [76]. Developing in vitro correlates of protection will require understanding of how infection modulates DC function and how these moving targets might be best eliminated, including at the olfactory mucosa. 

Differences exist between human and mouse placentation including how maternal: fetal blood exchange is organized. Such anatomical differences likely contribute to the failure of MCMV to transmit vertically. In contrast, rhesus and guinea pig placentation provide more authentic models of human pregnancy and their respective CMVs infect both the placenta and the neonate. Thus, natural transmission studies in the guinea pig and rhesus models will provide valuable preclinical models to evaluate vaccine protection against congenital infection. 

Natural models of CMV infection provide insight in virus–host interactions that cannot be achieved by in vitro or ex vivo investigations. Differences exist between species how common mechanisms of immune evasion and host control are articulated, but the models nonetheless provide a thematic framework that guides further investigations of HCMV spread and how infection might be controlled. Studies of MCMV dissemination have also revealed that blood-borne DC can come from peripheral tissues by recirculation, with HEV supporting bi-directional traffic. DC recirculation might explain how the MCMV load is maintained via periodic, stochastic reactivations in peripheral tissues. In addition, the capacity for recirculation suggests that DC may be re-used and molded by their experience, suggesting a form of peripheral immunological memory, and facilitating chronic inflammation disorders linked to HCMV infections. 

## Data Availability

Not applicable.
